# Supplementary material for: White matter microstructure in face and body networks predicts facial expression and body posture perception across development
Source: Hum Brain Mapp. 2023 Jan 20;44(6):2307–22. doi: 10.1002/hbm.26211 (PMC10028674; doi:10.1002/hbm.26211)
Supplement: Supplementary file 1 — DATA S1 Supporting Information [file HBM-44-2307-s001.docx]

**Supplementary Materials**

Supplementary Table 1 (S1): *Age and microstructure.*

Right hemisphere functionally-defined fibre tracts (FDFTs) of face and body networks*.*

* shaded boxes indicate significant correlation (significance threshold *p*<0.008)

|  | **OFA-FFA** | **FFA-ATL** | **pSTSface-ATL** | **EBA-FBA** | **FBA-ATL** | **pSTSbody-ATL** |
| --- | --- | --- | --- | --- | --- | --- |
| **FA** | r_s_= 0.032, *p*=0.85 | r_s_= 0.064, *p*=0.69 | r_s_= 0.133, *p*=0.42 | r_s_= -0.378, *p*=0.03 | r_s_= -0.119, *p*=0.48 | r_s_= 0.256, *p*=0.12 |
| **R1** | *r_s_= 0.623, p<0.0001 ** | *r_s_= 0.514, p=0.0006 ** | *r_s_= 0.61, p<0.0001 ** | *r_s_= 0.476, p=0.003 ** | *r_s_= 0.516, p=0.0009 ** | *r_s_= 0.544, p=0.0004 ** |
| **Ṧ_µ_** | *r_s_= 0.580, p=0.0003 ** | *r_s_= 0.492, p=0.001 ** | *r_s_= 0.631, p<0.0001 ** | *r_s_= 0.470, p=0.005 ** | r_s_= 0.363, *p*=0.03 | *r_s_= 0.709, p<0.0001 ** |

Control tracts within (ILF) and outside (CST) the visual system

|  | **ILF** | **CST** |
| --- | --- | --- |
| **FA** | r_s_= 0.235, *p*=0.13 | r_s_= 0.114, *p*=0.55 |
| **R1** | *r_s_= 0.65, p<0.0001 ** | r_s_= 0.332, *p*=0.03 |
| **Ṧ_µ_** | *r_s_= 0.563, p=0.0001 ** | *r_s_= 0.616, p<0.0001 ** |

Supplementary Table 2 (S2): *Facial expression perception and microstructure.*

Right hemisphere FDFTs of face and body networks*.*

* shaded boxes indicate significant correlation (significance threshold *p*<0.008)

|  | **OFA-FFA** | **FFA-ATL** | **pSTSface-ATL** | **EBA-FBA** | **FBA-ATL** | **pSTSbody-ATL** |
| --- | --- | --- | --- | --- | --- | --- |
| **FA** | *r_s_= 0.562, p=0.006 ** | r_s_= 0.077, *p*=0.70 | r_s_= 0.031, *p*=0.88 | r_s_= -0.147, *p*=0.48 | r_s_= 0.046, *p*=0.83 | r_s_= 0.336, *p*=0.09 |
| **R1** | *r_s_= 0.623, p=0.0019 ** | r_s_= 0.447, *p*=0.016 | r_s_= 0.270, *p=*0.16 | r_s_= 0.398, *p*=0.05 | r_s_= 0.376, *p*=0.06 | r_s_= 0.331, *p*=0.10 |
| **Ṧ_µ_** | *r_s_= 0.553, p=0.0069 ** | r_s_= 0.389, *p*=0.04 | r_s_= 0.183, *p=*0.35 | r_s_= 0.279, *p*=0.176 | r_s_= 0.504, *p*=0.01 | r_s_= 0.332, *p=*0.10 |

Control tracts within (ILF) and outside (CST) the visual system

|  | **ILF** | **CST** |
| --- | --- | --- |
| **FA** | r_s_= -0.034, *p*=0.86 | r_s_= 0.133, *p*=0.59 |
| **R1** | r_s_= 0.446, *p*=0.02 | r_s_= 0.413, *p*=0.03 |
| **Ṧ_µ_** | r_s_= 0.203, *p*=0.29 | r_s_= 0.336, *p=*0.08 |

Supplementary Table 3 (S3): *Body posture perception and microstructure.*

Right hemisphere FDFTs of face and body networks*.*

* shaded boxes indicate significant correlation (significance threshold *p*<0.008)

|  | **OFA-FFA** | **FFA-ATL** | **pSTSface-ATL** | **EBA-FBA** | **FBA-ATL** | **pSTSbody-ATL** |
| --- | --- | --- | --- | --- | --- | --- |
| **FA** | r_s_= 0.279, *p*=0.15 | r_s_= -0.250, *p*=0.17 | r_s_= 0.076, *p*=0.68 | r_s_= -0.272, *p*=0.15 | r_s_= -0.167, *p*=0.38 | r_s_= 0.193, *p*=0.29 |
| **R1** | *r_s_= 0.590, p=0.001 ** | r_s_= 0.355, *p*=0.04 | r_s_= 0.32, *p=*0.08 | r_s_= 0.375, *p*=0.04 | r_s_= 0.404, *p*=0.03 | r_s_= 0.359, *p*=0.04 |
| **Ṧ_µ_** | *r_s_= 0.597, p=0.001 ** | r_s_= 0.310, *p*=0.09 | r_s_= 0.265, *p=*0.14 | r_s_= 0.391, *p*=0.03 | r_s_= 0.325, *p*=0.08 | r_s_= 0.413, *p=*0.02 |

Control tracts within (ILF) and outside (CST) the visual system

|  | **ILF** | **CST** |
| --- | --- | --- |
| **FA** | r_s_= 0.004, *p*=0.98 | r_s_= 0.118, *p*=0.59 |
| **R1** | r_s_= 0.247, *p*=0.16 | r_s_= 0.202, *p*=0.25 |
| **Ṧ_µ_** | r_s_= 0.206, *p*=0.23 | r_s_= 0.334, *p=*0.05 |

Supplementary Table 4 (S4): *Influence of body posture on facial expression perception (PSE change) and microstructure.*

Right hemisphere FDFTs of face and body networks*.*

* shaded boxes indicate significant correlation (significance threshold *p*<0.008)

|  | **OFA-FFA** | **FFA-ATL** | **pSTSface-ATL** | **EBA-FBA** | **FBA-ATL** | **pSTSbody-ATL** |
| --- | --- | --- | --- | --- | --- | --- |
| **FA** | r_s_= -0.247, *p*=0.29 | r_s_= -0.196, *p*=0.36 | r_s_= -0.096, *p*=0.66 | r_s_= 0.312, *p*=0.15 | r_s_= -0.068, *p*=0.76 | r_s_= -0.061, *p*=0.78 |
| **R1** | r_s_= -0.517, *p=*0.02 | r_s_= -0.493, *p*=0.01 | r_s_= -0.47, *p=*0.03 | r_s_= -0.425, *p*=0.04 | r_s_= -0.387, *p*=0.07 | *r_s_= -0.593, p=0.002 ** |
| **Ṧ_µ_** | r_s_= -0.245, *p*=0.30 | r_s_= -0.383, *p*=0.07 | r_s_= -0.406, *p=*0.05 | r_s_= -0.008, *p*=0.97 | r_s_= -0.196, *p*=0.38 | *r_s_= -0.605, p=0.002 ** |

Control tracts within (ILF) and outside (CST) the visual system

|  | **ILF** | **CST** |
| --- | --- | --- |
| **FA** | r_s_= -0.265, *p*=0.19 | r_s_= 0.069, *p*=0.79 |
| **R1** | *r_s_= -0.592, p=0.002 ** | r_s_= -0.372, *p*=0.07 |
| **Ṧ_µ_** | r_s_= -0.441, *p*=0.03 | r_s_= -0.461, *p=*0.02 |

Multiple Regression Models to look at contribution of age and right hemisphere tract microstructure in predicting perception.

*significant at p<0.05

Supplementary Table 5 (S5):

*Facial Expression Perception ~ Age + FA_OFA-FFA_*

R^2^: 0.428

Adjusted R^2^: 0.371

F(2,20)= 7.50, *p*=0.004

|  | Coefficients | Standard Error | t statistic | *p*-value |
| --- | --- | --- | --- | --- |
| Intercept | -11.88 | 4.355 | -2.728 | 0.013 * |
| Age | 0.332 | 0.136 | 2.448 | 0.024 * |
| FA_OFA-FFA_ | 28.21 | 11.42 | 2.470 | 0.023 * |

*Variance Inflation Factor (VIF): 1.04*

Supplementary Table 6 (S6):

*Facial Expression Perception ~ Age + R1_OFA-FFA_*

R^2^: 0.432

Adjusted R^2^: 0.375

F(2,20)= 7.61, *p*=0.003

|  | Coefficients | Standard Error | t statistic | *p*-value |
| --- | --- | --- | --- | --- |
| Intercept | -30.75 | 11.60 | -2.651 | 0.015 * |
| Age | 0.089 | 0.181 | 0.492 | 0.628 |
| R1_OFA-FFA_ | 39.73 | 15.86 | 2.505 | 0.021 * |

*VIF: 1.86*

Supplementary Table 7 (S7):

*Facial Expression Perception ~ Age + Ṧ_µ_ _OFA-FFA_*

R^2^: 0.313

Adjusted R^2^: 0.245

F(2,20)= 4.56, *p*=0.023

|  | Coefficients | Standard Error | t statistic | *p*-value |
| --- | --- | --- | --- | --- |
| Intercept | -8.621 | 5.381 | -1.602 | 0.125 |
| Age | 0.231 | 0.193 | 1.199 | 0.245 |
| Ṧ_µ OFA-FFA_ | 69.06 | 52.65 | 1.312 | 0.204 |

*VIF: 1.75*

Supplementary Table 8 (S8):

*Body Posture Perception ~ Age + R1_OFA-FFA_*

R^2^: 0.347

Adjusted R^2^: 0.295

F(2,25)= 6.64, *p*=0.005

|  | Coefficients | Standard Error | t statistic | *p*-value |
| --- | --- | --- | --- | --- |
| Intercept | -19.22 | 15.43 | -1.246 | 0.224 |
| Age | 0.446 | 0.256 | 1.746 | 0.093 |
| R1 _OFA-FFA_ | 23.79 | 21.39 | 1.112 | 0.277 |

*VIF: 1.91*

Supplementary Table 9 (S9):

*Body Posture Perception ~ Age + Ṧ_µ_ _OFA-FFA_*

R^2^: 0.366

Adjusted R^2^: 0.315

F(2,25)= 7.21, *p*=0.003

|  | Coefficients | Standard Error | t statistic | *p*-value |
| --- | --- | --- | --- | --- |
| Intercept | -9.83 | 5.86 | -1.678 | 0.106 |
| Age | 0.435 | 0.234 | 1.858 | 0.075 |
| Ṧ_µ OFA-FFA_ | 81.96 | 57.82 | 1.417 | 0.169 |

*VIF: 1.65*

Supplementary Table 10 (S10):

*Influence of body posture on facial expression perception ~ Age + R1_STSb-ATL_*

R^2^: 0.351

Adjusted R^2^: 0.290

F(2,21)= 5.69, *p*=0.011

|  | Coefficients | Standard Error | t statistic | *p*-value |
| --- | --- | --- | --- | --- |
| Intercept | 1.83 | 0.672 | 2.726 | 0.013 * |
| Age | -0.006 | 0.010 | -0.583 | 0.566 |
| R1 _STSb-ATL_ | -1.92 | 0.903 | -2.126 | 0.046 * |

*VIF: 1.75*

Supplementary Table 11 (S11):

*Influence of body posture on facial expression perception ~ Age + Ṧ_µ_ _STSb-ATL_*

R^2^: 0.289

Adjusted R^2^: 0.221

F(2,21)= 4.27, *p*=0.028

|  | Coefficients | Standard Error | t statistic | *p*-value |
| --- | --- | --- | --- | --- |
| Intercept | 1.26 | 0.565 | 2.226 | 0.037 * |
| Age | -0.007 | 0.012 | -0.546 | 0.591 |
| Ṧ_µ STSb-ATL_ | -7.71 | 5.11 | -1.510 | 0.146 |

*VIF: 2.41*

Supplementary Table 12 (S12):

*Influence of body posture on facial expression perception ~ Age + R1_ILF_*

R^2^: 0.332

Adjusted R^2^: 0.271

F(2,22)= 5.46, *p*=0.011

|  | Coefficients | Standard Error | t statistic | *p*-value |
| --- | --- | --- | --- | --- |
| Intercept | 2.01 | 0.795 | 2.531 | 0.019 * |
| Age | -0.006 | 0.010 | -0.621 | 0.541 |
| R1 _ILF_ | -2.15 | 1.06 | -2.032 | 0.054 |

*VIF: 1.77*
